# Synthesis and Guest-Binding Properties of pH/Reduction Dual-Responsive Cyclophane Dimer

**DOI:** 10.3390/molecules26113097

**Published:** 2021-05-22

**Authors:** Osamu Hayashida, Yudai Tanaka, Takaaki Miyazaki

**Affiliations:** Department of Chemistry, Faculty of Science, Fukuoka University, Nanakuma 8-19-1, Fukuoka 814-0180, Japan; sd192007@cis.fukuoka-u.ac.jp (Y.T.); t.miyazaki@fukuoka-u.ac.jp (T.M.)

**Keywords:** cyclophane dimer, host-guest complexation, pH-dependent guest-binding, reduction-responsive guest release

## Abstract

A water-soluble cyclophane dimer having two disulfide groups as a reduction-responsive cleavable bond as well as several acidic and basic functional groups as a pH-responsive ionizable group **1** was successfully synthesized. It was found that **1** showed pH-dependent guest-binding behavior. That is, **1** strongly bound an anionic guest, 6-*p*-toluidinonaphthalene-2-sulfonate (TNS) with binding constant (*K*/M^−1^) for 1:1 host-guest complexes of 9.6 × 10^4^ M^−1^ at pH 3.8, which was larger than those at pH 7.4 and 10.7 (6.0 × 10^4^ and 2.4 × 10^4^ M^−1^, respectively), indicating a favorable electrostatic interaction between anionic guest and net cationic **1**. What is more, release of the entrapped guest molecules by **1** was easily controlled by pH stimulus. Large favorable enthalpies (Δ*H*) for formation of host-guest complexes were obtained under the pH conditions employed, suggesting that electrostatic interaction between anionic TNS and **1** was the most important driving force for host-guest complexation. Such contributions of Δ*H* for formation of host-guest complexes decreased along with increased pH values from acidic to basic solutions. Upon addition of dithiothreitol (DTT) as a reducing reagent to an aqueous PBS buffer (pH 7.4) containing **1** and TNS, the fluorescence intensity originating from the bound guest molecules decreased gradually. A treatment of **1** with DTT gave **2**, having less guest-binding affinity by the cleavage of disulfide bonds of **1**. Consequently, almost all entrapped guest molecules by **1** were released from the host. Moreover, such reduction-responsive cleavage of **1** and release of bound guest molecules was performed more rapidly in aqueous buffer at pH 10.7.

## 1. Introduction

Synthetic macrocyclic hosts play a crucial role in supramolecular chemistry and nanoscience [1,2,3,4]. Currently, the development of a stimuli-responsive supramolecular system [5,6] has been attracting much attention for the purpose of bio-molecular sensing [7,8,9,10] and drug delivery systems [11,12]. Particularly, water-soluble macrocyclic hosts based on cucurbit[n]urils [13,14,15], calix[n]arenes [16,17,18,19], pillar[n]arenes [20,21], cyclophanes [22,23,24,25,26], and others have been frequently used for such purposes and numerous applications. For instance, Isaacs et al. reported that the cucurbit[n]uril family acted as synthetic hosts to give rise to a controlled guest binding and release in response to a change in pH [27]. Lee et al. developed attractive approaches to produce stimuli-responsive supramolecular nanocapsules based on amphiphilic calix[n]arenes to trigger a release of the encapsulated hydrophobic guest molecules [28]. Recently, stimuli-responsive nano-sized assemblies based on water-soluble pillar[n]arene in the potential applications of drug delivery systems were reviewed by Wang et al. [29].

**Figure 1:**
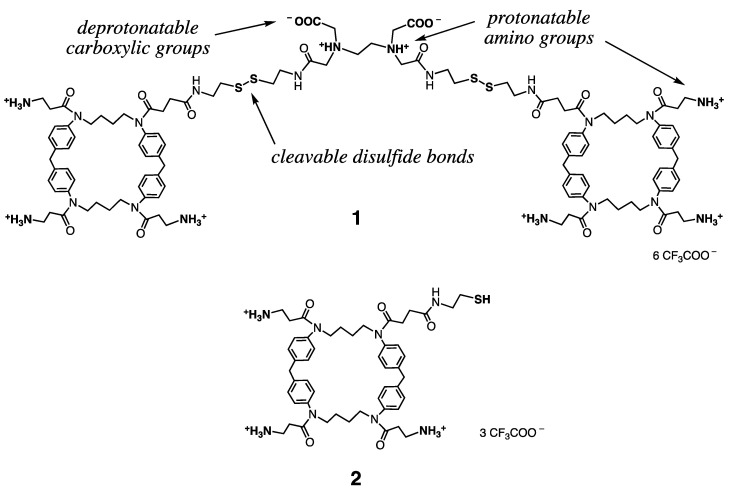
pH/reduction-responsive cyclophane dimer **1** and corresponding monomeric cyclophane **2**.

**Figure 2:**
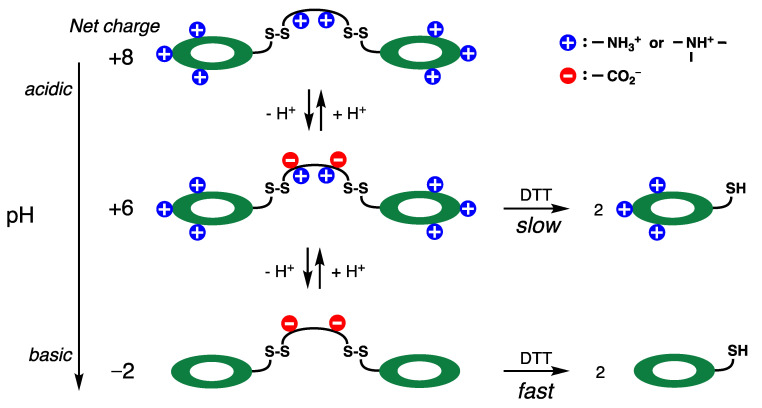
Schematic representation for protonation and deprotonation equilibria in the pH-responsive cyclophane dimer **1** and the transformation to the corresponding monomeric cyclophane thiol **2** by reductants, such as dithiothreitol (DTT).

Among these hosts, azacyclophanes have an advantage from the view point of their modifications by attaching various functional moieties on the nitrogen atoms of the macrocyclic skeleton [30,31,32]. Our effort has been paid to functionalize the tetraaza [6.1.6.1]paracyclophane skeleton, which can act as a guest-binding site [33,34]. As is well known, a disulfide bond is a reduction-responsive and cleavable connector [35,36,37]. In the preceding paper, we reported a reduction-responsive water-soluble cyclophane dimer having a disulfide linkage [38]. The guest-binding affinity of the cyclophane dimer was found to be increased relative to that by the corresponding monocyclic cyclophane [38]. Reduction of the disulfide bond of the cyclophane dimer by reducing reagents gave monocyclic cyclophanes having less guest-binding affinity to release the entrapped guest molecules [38]. Recently, we also developed reduction-responsive supramolecular host-guest aggregation/disaggregation systems based on disulfide linkages [39]. In the next strategy of our research on the stimuli-responsible water-soluble cyclophane dimers, we became interested in developing pH/reduction dual-responsive hosts to increase the responsivity to external stimuli for the guest capture and release systems. Here, we now report the design and synthesis of water-soluble cyclophane dimer bearing different moieties responsive to two different external stimuli **1** (Figure 1). The former is reduction-responsive cleavable disulfide bonds while the latter is several acidic and basic functional groups as a pH-responsive ionizable group. Total net charges of **1** are designed to decrease by changing pH from acidic to basic aqueous solutions, which reflect differences in their guest capture and release abilities (Figure 2). Host **1** having disulfide bonds is expected to be broken down into the corresponding monomeric cyclophanes **2** by reductants, such as dithiothreitol (DTT). In addition, we demonstrated pH-dependent guest-binding behavior and reduction-responsive cleavage of **1**, with a concomitant enhanced release of bound guest molecules.

## 2. Results and Discussion

### 2.1. Design and Synthesis of pH/Reduction-Responsive Cyclophane Dimer

We designed a water-soluble cyclophane dimer bearing two disulfide groups as a reduction-responsive cleavable bond as well as several acidic and basic functional groups as a pH-responsive ionizable group **1** (Figure 1). Cyclophane dimer **1** was constructed with two tetraaza[6.1.6.1]-paracyclophanes [40] and a zwitterionic ionizable linker connected with disulfide bonds. Each former cyclophane skeleton has a hydrophobic cavity for guest binding and three hydrophilic polar side chains with an ammonium group. The latter linker originating from ethylenediaminetetraacetic dianhydride (EDTAD) affords deprotonatable carboxylic acids and protonatable amino groups. The carboxylic acid is uncharged below its p*K*a, while it is deprotonated and thus the charged form is above the p*K*_a_ [41]. Similarly, the peripheral primary and central tertiary ammonium groups also act as pH-responsive ionizable groups depending on their p*K*_a_ [41]. That is, compound **1** is expected to act as a water-soluble host in aqueous buffer over a wide pH range due to the ionizable polar side chains and linker. In addition, the net cationic charge of compound **1** is expected to decrease by changing pH from acidic to basic aqueous solutions, as shown in Figure 2.

**Scheme 1:**
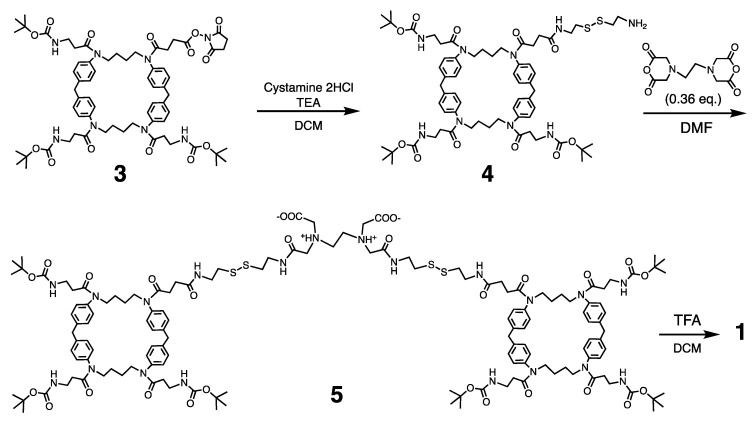
Preparation of pH/reduction-responsive cyclophane dimer **1**.

Cyclophane dimer **1** was synthesized as shown in Scheme 1. In the previous study, we reported a synthesis of a succinimidyl ester derivative of cyclophane having three Boc-protected β-alanine residues **3** [42]. A cyclophane derivative tethered with cystamine **4** was prepared by a reaction of **3** with cystamine dihydrochloride in the presence of triethylamine. A precursor (**5**) of **1** was synthesized by aminolysis of **4** with EDTAD. A treatment with trifluoroacetic acid (TFA) gave water-soluble cyclophane dimer **1** from **5** by removal of the protecting groups in a fairly good yield. All the new obtained compounds were fully assigned by ^1^H and ^13^C NMR spectroscopy, matrix-assisted laser desorption time of flight mass spectrometry (MALDI-TOF MS), and elemental analysis (see the Supplementary Materials). Despite the presence of a hydrophobic macrocyclic skeleton, compound **1** has high solubility in a wide pH range of aqueous media owing to the ionizable polar side chains and linker.

### 2.2. pH-Responsive Guest-Binding Behavior of **1**

**Figure 3:**
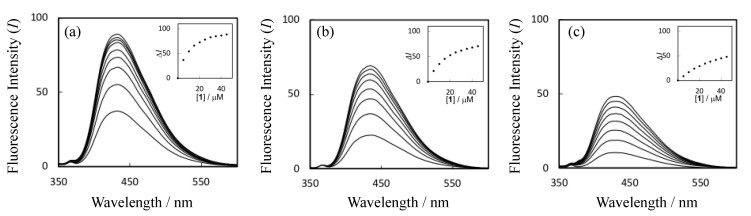
Fluorescence spectra of TNS (1.0 μM) in aqueous buffer at pH 3.8 (**a**), 7.4 (**b**), and 10.7 (**c**) upon addition of **1** at 298 K. [1] = 0, 5, 10, 15, 20, 25, 30, 35, 40, and 45 μM. Ex. 326 nm.

**Figure 4:**
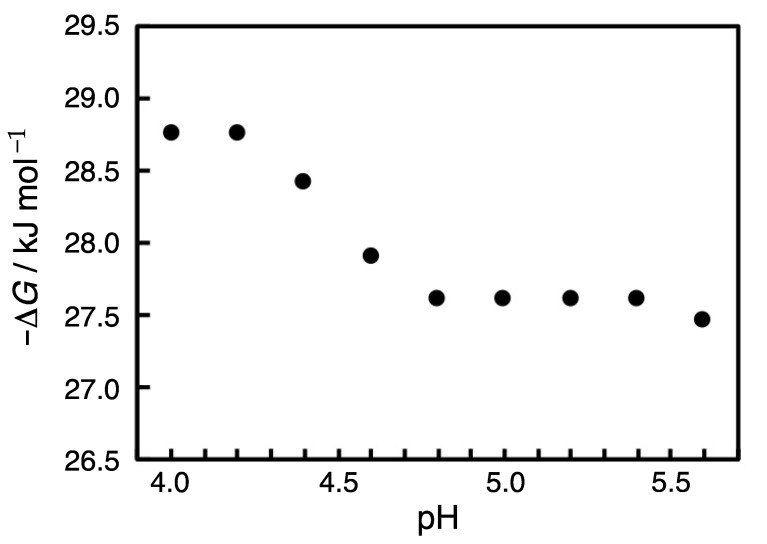
Correlation of Gibbs free energy (Δ*G*) for formation of host-guest complexes with pH values.

pH-dependent guest-binding ability of **1** toward 6-*p*-toluidinonaphthalene-2-sulfonate (TNS) as an anionic fluorescent guest, whose emission is extremely sensitive in the intensity to change in microenvironmental polarity surrounded by the molecule, was examined by fluorescence titration experiments at 298 K. First, we executed the fluorescence titration experiments of **1** with TNS at three pH levels: 3.8 (acidic), 7.4 (neutral), and 10.7 (basic). Upon addition of **1** in a large excess amount to each of the aqueous buffers containing TNS, the fluorescence intensity originated from the entrapped guest increased, showing saturation behavior, as shown in Figure 3. The extent of saturated changes in the emission intensity of TNS by the complexation with **1** increased in the following sequence of pH: 3.8 > 7.4 > 10.7 (Figure 3). The binding constant (*K*/M^−1^) for 1:1 host-guest complexes was calculated on the basis of the Benesi–Hildebrand method [43] for the titration data. It was found that **1** showed pH-dependent guest-binding behavior. That is, the *K* value of **1** with TNS at pH 3.8 (9.6 × 10^4^ M^−1^) and was larger than those at pH 7.4 and 10.7 (6.0 × 10^4^ and 2.4 × 10^4^ M^−1^, respectively), suggesting a favorable electrostatic interaction between anionic guest and net cationic **1**. Then, in order to investigate the pH-dependent guest-binding behavior, we tried to evaluate the binding behavior of **1** with TNS in detail at a pH ranging between 4.0 and 5.6 by the identical method. A plot of the correlation of the Gibbs free energy (Δ*G*) for formation of host-guest complexes with pH values is shown in Figure 4, indicating an equivalence point, which was equal to p*K*a of carboxylic acids of **1**. The p*K*_a_ value of the carboxylic acids of **1** was estimated to be ca. 4.5, which was generally close to the values of acetic acid (p*K*_a_, 4.76) [41] or propanoic acid (p*K*_a_, 4.87) [41]. Because the carboxylic acids are uncharged below the p*K*_a_, compound **1** has the net cationic charge (+8), as shown in Figure 2. On the other hand, the carboxylic acids are deprotonated and thus the charged form above the p*K*_a_, compound **1**, has the net cationic charge (+6), as shown in Figure 2. Therefore, the electrostatic interaction between anionic TNS and **1** below the p*K*_a_ was more favored than the corresponding interaction above the p*K*_a_. It was found that **1** showed pH-dependent guest-binding behavior, indicating the net cationic charge of compound **1** is the most important for host–guest interaction. Despite host **1** being in its anionic form at pH 10.7, which causes electrostatic repulsion with the anionic guest, it still binds anionic TNS in aqueous media. Hence, hydrophobic interaction between **1** and TNS is considered to be one of the important driving forces.

**Figure 5:**
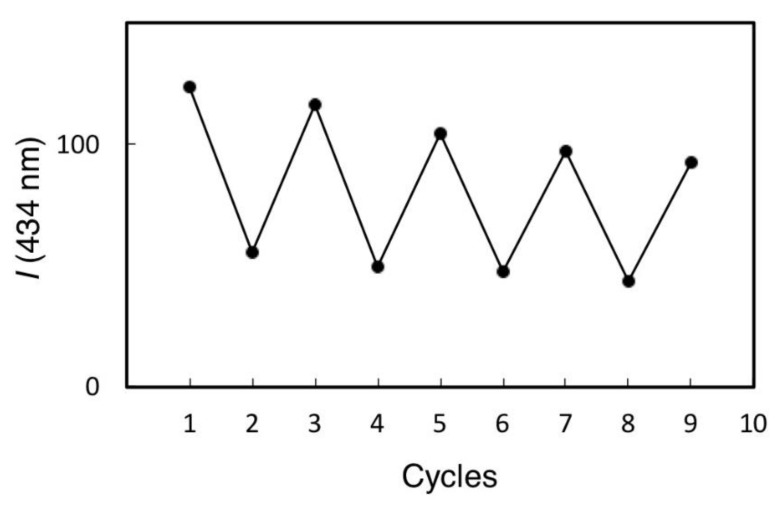
Reversibility study on the fluorescence cycle for complexation of **1** with TNS using NaOH and HCl solutions. Ex. 326 nm.

Then, we investigated reversible changes of the fluorescence spectra in the intensity of TNS in the presence of **1** at different pH tuned by NaOH and HCl. Upon addition of NaOH to aqueous buffers containing **1** and TNS, the fluorescence intensity originated from TNS decreased, as shown in Figure 5. Moreover, the fluorescence intensity recovered, followed by addition of HCl to the alkaline solution (Figure 5). These fluorescence spectral changes induced by pH stimulus were reversible and can be repeated several times (Figure 5), even though the extent of change in fluorescence intensity tended to be smaller, owing to the diluted solution and formation of NaCl during the acid-base circle. These results indicate that the binding and release of the guest by **1** was easily controlled by pH stimulus.

### 2.3. Thermodynamically Characterization of Guest-Binding Behavior of **1**

pH-responsible cyclophane dimer **1** binds TNS through electrostatic and hydrophobic interactions, as mentioned above. In order to consider the host–guest interactions in all, we tried to evaluate thermodynamic parameters from temperature-dependent *K* values for **1** with TNS. Consequently, we executed the fluorescence titration experiments of **1** with TNS at 288, 298, 308, and 318 K at three pH levels: 3.8 (acidic), 7.4 (neutral), and 10.7 (basic) (Figure S5). The obtained *K* values are summarized in Table 1. In addition, Gibbs free energy (Δ*G*), enthalpies (Δ*H*), and entropies (Δ*S*) for the formation of host-guest complexes, which were evaluated from Van’t Hoff analysis (Figure S6), are summarized in Table 2. As is obvious from the data in Table 2, large favorable Δ*H* values were obtained under the pH conditions employed. These results suggest that electrostatic interaction between anionic TNS and **1** was the most important driving force for host-guest complexation. Such contributions of Δ*H* values on the host-guest complexation decreased along with increased pH values from acidic to basic solutions (Table 2).

### 2.4. Reduction-Responsive Guest-Binding Behavior of **1**

**Figure 6:**
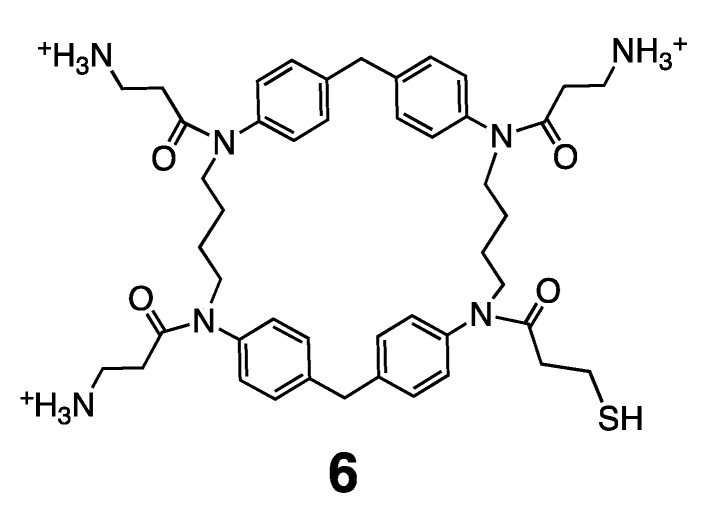
Monocyclic cyclophane having a thiol group.

Prior to the reduction-responsive guest binding/releasing behavior of **1**, multivalent effects on the guest-binding in macrocycles were examined. Herein, we compared control compound **6** [38] as a monocyclic cyclophane having a thiol group (Figure 6). We reported previously that the *K* value of **6** with TNS [38], which was obtained by fluorescence titration experiments in the presence of DTT in an aqueous buffer (pH 7.4) at 298 K, was relatively small (*K* = 9.6 × 10^3^ M^−1^). Therefore, the *K* value of cyclophane dimer **1** with the identical guest was enhanced about 6-fold relative to those of **6**, reflecting multivalent effects in macrocycles.

Controlled binding/releasing of guest molecules by stimuli-responsive hosts is an attractive research topic. In regard to the reduction-responsive host-guest chemistry, the present cyclophane dimer has two disulfide moieties that are cleavable to thiols by a treatment with reductants. Actually, **1** was easily transformed to the corresponding monocyclic cyclophane thiol **2** by reducing reagents, such as DTT. In a proof-of-principal experiment, degradation of **1** to thiols by DTT was verified by MALDI-TOF MS spectrometry. That is, upon addition of DTT to aqueous PBS buffer (pH7.4) of **1**, a peak originating from a reduced form of **1**, a thiol derivative of cyclophane **2**, was observed, i.e., *m/z*, 899.7 was assigned to [M + Na]^+^, where M stands for the corresponding thiol of cyclophane, C_49_H_64_N_8_O_5_S (Figure S7).

**Figure 7:**
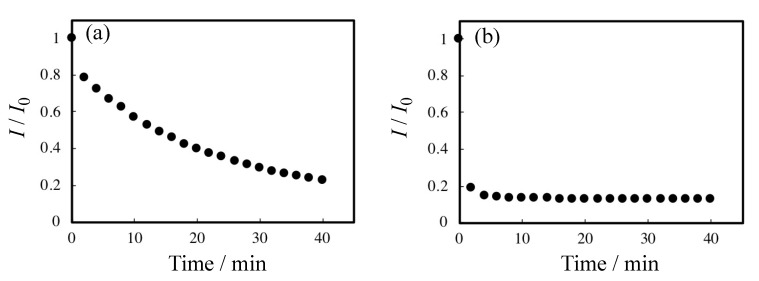
Time course of the fluorescence intensity change of entrapped TNS (1.0 μM) in the presence of **1** (25 μM) upon addition of DTT (50 μM) in aqueous buffer at pH 7.4 (**a**) and 10.7 (**b**).

Then, the controlled release of guest molecules by **1** was studied by fluorescence spectroscopy. Upon addition of DTT to a PBS buffer (pH 7.4) containing **1** and TNS at 298 K, the fluorescence intensity originating from the bound guest molecules decreased gradually, as shown in Figure 7a. A treatment of **1** with DTT gave **2** with less guest-binding affinity by the cleavage of disulfide bonds of **1**. Accordingly, almost all entrapped guest molecules by **1** were released to the bulk aqueous phase. Moreover, reduction-responsive cleavage of **1**, accompanying the release of entrapped guest molecules, was performed more rapidly in aqueous buffer at pH 10.7, as shown in Figure 7b. These results indicate that the nucleophilic reactivity of DTT with a disulfide group increases at an alkaline pH. On the other hand, such release of the entrapped guest hardly occurred at acidic pH, due to the poor nucleophilic reactivity of DTT. Similarly, upon addition of glutathione (GSH) to a PBS buffer solution containing host-guest complexes of **1** with TNS, the fluorescence intensity decreased, indicating the release of the entrapped guest molecules (Figure S8).

## 3. Experimental Section

### 3.1. Materials

Succinimidyl ester derivative of tetraaza[6.1.6.1]paracyclophane bearing Boc-protected β-alanine residues (**3**) was prepared according to the literature reported previously [42]. 6-*p*-toluidinonaphthalene-2-sulfonate (TNS) was obtained from a commercial source from Sigma-Aldrich (St. Louis, MO, USA) and used without further purification. Acetate buffer (pH 3.8–4.8), phosphate buffer (pH 5.0–7.0), 2-[4-(2-hydroxyethyl)-1-piperazinyl]ethanesulfonic acid (HEPES) buffer (pH 7.4), and carbonate buffer (pH 10.7) were used for fluorescence experiments.

### 3.2. Cyclophane Derivative Tethered with Cystamine (**4**)

A solution of succinimidyl ester derivative of cyclophane (**3**) [42] (0.20 g, 0.15 mmol) in dry dichloromethane (DCM, 15 mL) was added dropwise to a solution of cystamine dihydrochloride (0.70 g, 3.1 mmol) and triethylamine (2.0 mL) in dry DCM (10 mL), and the mixture was stirred for 12 h at room temperature. The solvent was evaporated on a rotatory evaporator to give a white solid. The crude product was purified on a column of Sephadex LH-20 (methanol) for purification. The main fraction was evaporated on a rotatory evaporator and dried under reduced pressure to give a white solid (190 mg, 92%): ^1^H NMR (400 MHz, CDCl_3_, 298 K) δ 1.44 (m, 35H), 2.19 (m, 6H), 2.36 (t, 2H), 2.55 (t, 2H), 3.02 (t, 2H), 3.23 (t, 2H), 3.29 (m, 6H), 3.53 (m, 2H), 3.64 (m, 10H), 3.97 (s 4H), 5.35 (s, 3H), 7.00 (m, 8H), and 7.22 (m, 8H). ^13^C NMR (100 MHz, CDCl_3_, 298 K): δ 24.8, 25.4, 26.1, 28.4, 29.5, 30.5, 30.6, 32.3, 33.2, 34.8, 36.4, 41.1, 48.8, 48.9, 70.0, 128.3, 130.2, 140.3, 140.5, 154.4, 155.9, 170.6, and 171.9. IR: 1635, 1701 cm^−1^ (C=O). Found: C, 58.10; H, 7.76; N, 9.21. Calcd for C_66_H_93_N_9_O_11_S_2_•6H_2_O: C, 58.26; H, 7.78; N, 9.26. MALDI-TOF MS *m*/*z*: 1274.9 [M + Na]^+^, where M shows C_66_H_93_N_9_O_11_S_2_.

### 3.3. Cyclophane Dimer **1**

Ethylenediaminetetraacetic dianhydride (EDTAD, 11 mg, 0.04 mmol) was added to a solution of **4** (140 mg, 0.11 mmol) in dry *N, N*-dimethylformamide (DMF, 3 mL), and the resulting mixture was stirred for 5 days at 60 °C. Chloroform (100 mL) was added to the residue, and the mixture was then washed with saturated aqueous sodium chloride (20 mL). After being dried (Na_2_SO_4_), the solution was evaporated to dryness under reduced pressure to give a white solid. The crude product was purified on a column of Sephadex LH-20 (methanol) as an eluent for purification. Evaporation of the main fraction on a rotatory evaporator gave a white solid (**5**, 70 mg, 48%). Subsequently, trifluoroacetic acid (1.3 mL) was added to a solution of **5** (60 mg, 0.027 mmol) in dry DCM (4 mL), and the mixture was stirred for 3 h at room temperature. Evaporation of the solvent under reduced pressure gave a white solid. The crude product was purified on a column of Sephadex LH-20 (methanol) as an eluant. The product fraction was evaporated and dried in vacio to give a white solid. (40 mg, 85%): ^1^H NMR (400 MHz, CD_3_OD, 298 K) δ 1.44 (m, 16H), 2.05 (m, 4H), 2.15 (m, 4H), 2.35 (m, 4H), 2.44 (m, 8H), 2.82 (m, 4H), 2.89 (m, 4H), 3.07 (m, 4H), 3.12 (m, 8H), 3.20 (m, 4H), 3.45 (m, 4H), 3.58 (m, 8H), 3.69 (m, 20H), 4.04 (s, 8H), 6.92 (m, 8H), 7.18 (m, 8H), and 7.40 (m, 16H). ^13^C NMR (100 MHz, CD_3_OD, 298 K) δ 22.3, 22.4, 25.6, 28.7, 31.0, 34.5, 35.7, 40.3, 42.0, 47.6, 55.1, 121.8, 128.2, 130.2, 139.4, 141.5, 160.1, and 170.1. Found: C, 49.32; H, 6.16; N, 9.38. Calcd for C_124_H_156_F_18_N_20_O_28_S_4_•10H_2_O: C, 49.23; H, 5.86; N, 9.26. MALDI-TOF MS: *m*/*z* 2162.3 [M + H]^+^ and 2184.3 [M + Na]^+^, where M denotes cyclophane derivative as a free base and carboxylic acid (C_112_H_150_N_20_O_16_S_4_).

### 3.4. Binding Constants of Cyclophane with TNS

To each solution of TNS (1.0 μM), HEPES buffer (0.01 M, pH 7.4, 0.15 M with NaCl) were added increasing amounts of **1**, and the fluorescence intensity originating fron the guest was monitored after each addition. The *K* values were calculated by using the Benesi–Hildebrand method for titration data.

### 3.5. General Measurements

Elemental analyses were measured using an elemental analyzer J-Science Lab JM11 (Kyoto, Japan). Fluorescence spectra, IR spectra, MALDI-TOF MS, and NMR spectra were recorded on a JASCO FP-750 (Tokyo, Japan), Perkin-Elmer spectrum one spectrometer (Waltham, MA, USA), Bruker autoflex speed (Billerica, MA, USA), and Bruker Avance III 400 (Billerica, MA, USA), respectively.

## 4. Conclusions

We synthesized a water-soluble cyclophane dimer with two disulfide groups as a reduction-responsive cleavable bond as well as several acidic and basic functional groups as a pH-responsive ionizable group **1**. Host **1** was found to show pH-dependent guest-binding behavior. The bind and release of TNS as an anionic guest by **1** was easily controlled by pH stimulus. Large favorable Δ*H* values were obtained under the pH conditions employed, suggesting that electrostatic interaction between anionic TNS and **1** was the most effective driving force for host-guest complexation. In addition, **1** was found to show reduction-responsive guest-binding behavior. Monomeric cyclophane **2** with less guest-binding affinity formed by the cleavage of disulfide bonds of **1** by DTT. Consequently, almost all entrapped guest molecules were released from **1**. Moreover, release of entrapped guest molecules proceeded more rapidly through reduction-responsive cleavage of **1** at basic pH. Finally, guest-binding and releasing abilities of **1** are controlled by pH stimulus as well as reducing reagents, such as DTT.

## Figures and Tables

**Table 1:** The binding constant (*K*/M^−1^) of **1** with TNS at 288, 298, 308, and 318 K at three pH levels: 3.8, 7.4, and 10.7.

	*T*/K			
pH	288	298	308	318
3.8	1.6 × 10^5^	9.6 × 10^4^	4.9 × 10^4^	2.4 × 10^4^
7.4	1.0 × 10^5^	6.0 × 10^4^	4.0 × 10^4^	2.3 × 10^4^
10.7	3.3 × 10^4^	2.4 × 10^4^	1.7 × 10^4^	1.2 × 10^4^

**Table 2:** Free energy change (Δ*G*, kJ mol^−1^) and thermodynamic parameters of enthalpies (Δ*H*, kJ mol^−1^) and entropies (Δ*S*, kJ mol^−1^ K^−1^) for formation of host-guest complexes at 298 K at pH of 3.8, 7.4, and 10.7.

pH	Δ*G*, kJ mol^−1^	Δ*H*, kJ mol^−1^	*T*Δ*S*, kJ mol^−1^
3.8	−28.4	−75.2	−46.8
7.4	−27.2	−64.3	−37.1
10.7	−25.0	−49.9	−24.9

## Supplementary Materials

The following are available online, Figure S1: 1H NMR spectrum of compound **4**. Figure S2: 13C NMR spectrum of compound **4**. Figure S3: 1H NMR spectrum of compound **1**. Figure S4: 13C NMR spectrum of compound **1**. Figure S5: Fluorescence titration spectra at 288, 298, 308, 318K. Figure S6: Van’t Hoff analysis. Figure S7: MALDI-TOF MS spectra of **1** in the presence of DTT. Figure S8: Time course for changes fluorescence upon addition of GSH.

## Sample Availability

Samples of the compounds are not available from authors.
