# Supplementary material for: Synthesis and Guest-Binding Properties of pH/Reduction Dual-Responsive Cyclophane Dimer
Source: Molecules. 2021 May 22;26(11):3097. doi: 10.3390/molecules26113097 (PMC8196905; doi:10.3390/molecules26113097)
Supplement: Supplementary file 1 [file molecules-26-03097-s001.zip › molecules-1221702-supplementary.pdf]

Supplementary Materials

Synthesis and Guest-binding Properties of pH/Reduction Dual-Responsive Cyclophane Dimer

Osamu Hayashida Yudai Tanaka, and Takaaki Miyazaki*

Department of Chemistry, Faculty of Science, Fukuoka University, 8-19-1 Nanakuma, Fukuoka 814-0180, Japan

Table of Contents **Page:**

Fig. S1. ^{1}H NMR spectrum of compound **4** S2
Fig. S2. ^{13}C NMR spectrum of compound **4** S3
Fig. S3. ^{1}H NMR spectrum of compound **5** S4
Fig. S4. ^{1}H NMR spectrum of compound **1** S5
Fig. S5. ^{13}C NMR spectrum of compound **1** S6
Fig. S6. Fluorescence titration spectra at 288, 298, 308, 318K S7
Fig. S6 (continued). Double-reciprocal plots S8
Fig. S7. van't Hoff analysis S9
Fig. S8. MALDI-TOF MS spectra of **1** in the presence of DTT S10
Fig. S9. Time course for changes fluorescence upon addition of GSH S11

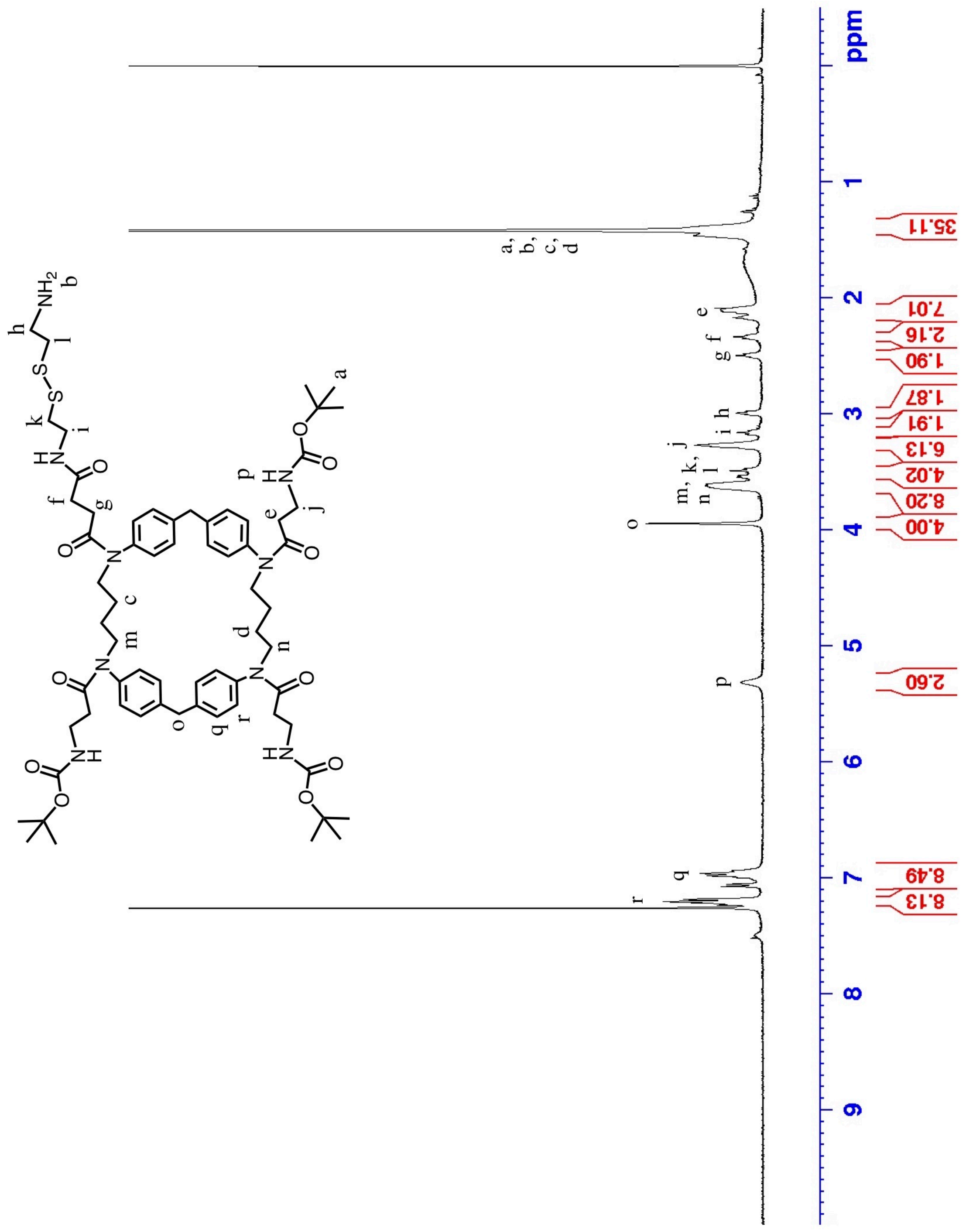

Figure S1. ^{1}H NMR spectrum of compound **4** (400 MHz, $CDCl_3$, 298K).

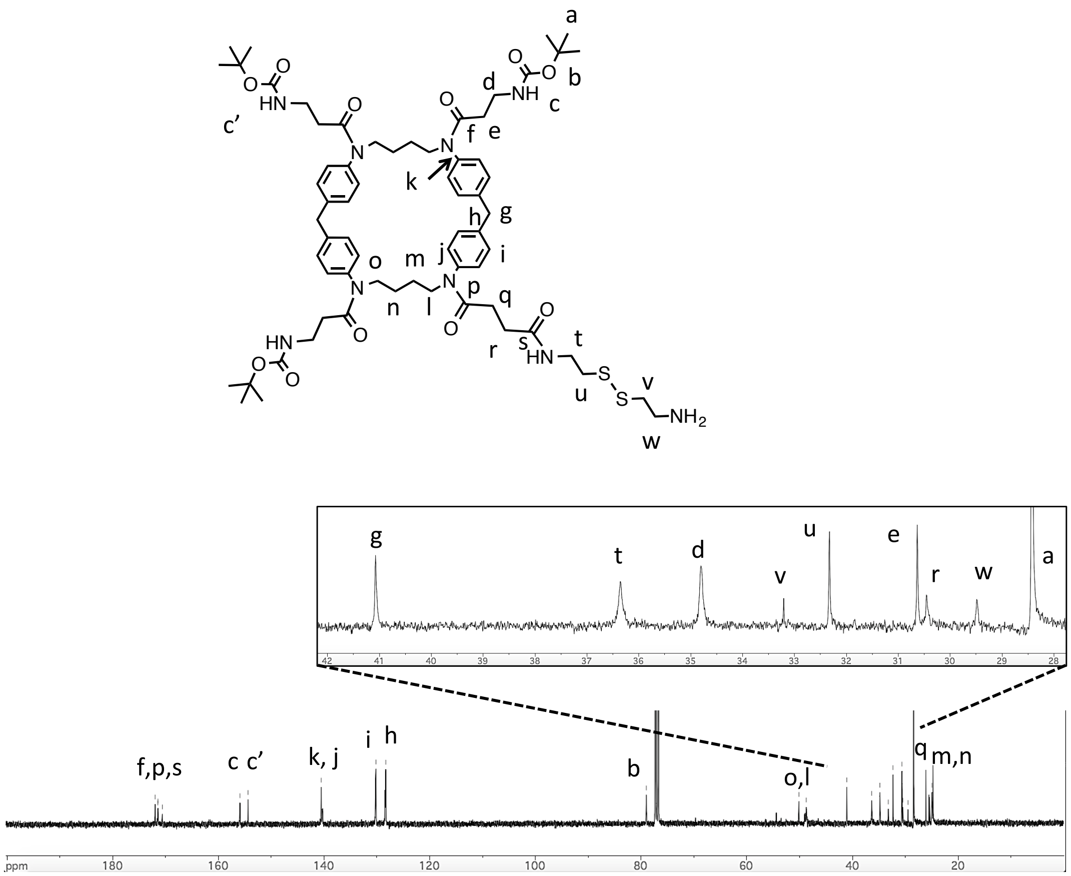

Figure S2. ^{13}C NMR spectrum of compound **4** (100 MHz, $CDCl_3$, 298K).

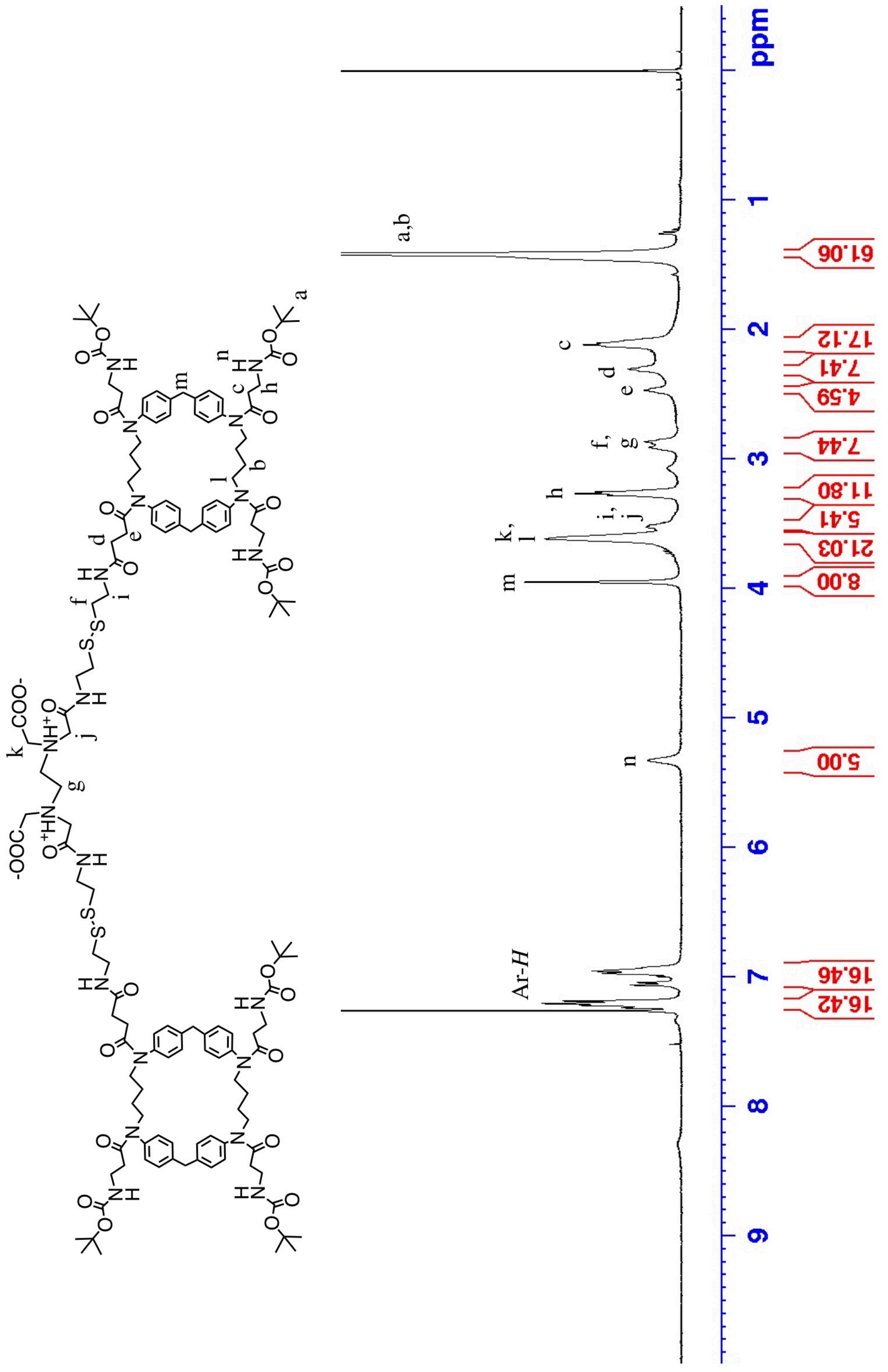

Figure S3. ^{1}H NMR spectrum of compound **5** (400 MHz, $CDCl_3$, 298K).

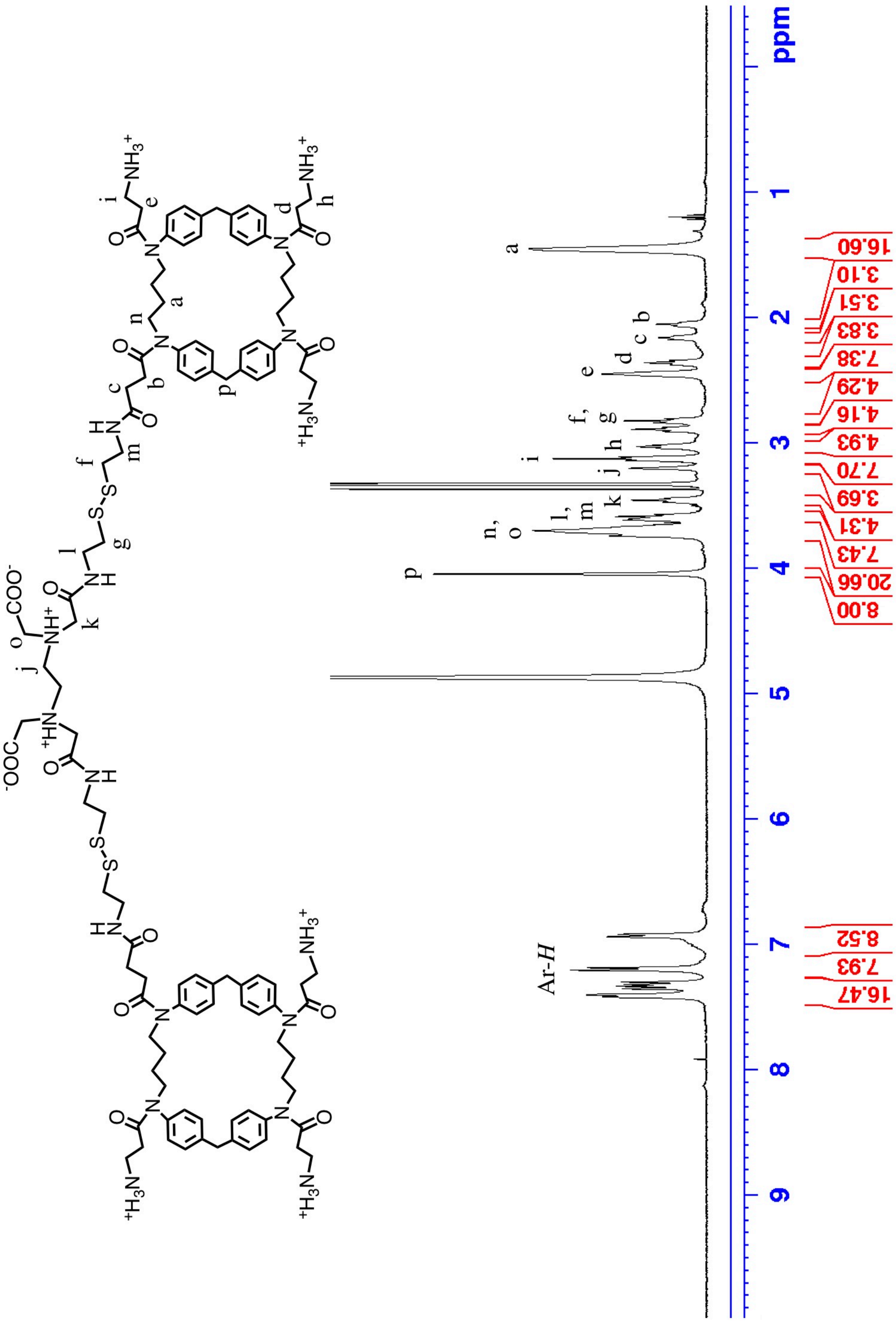

Figure S4. ^{1}H NMR spectrum of compound **1** (400 MHz, CD_3OD, 298K).

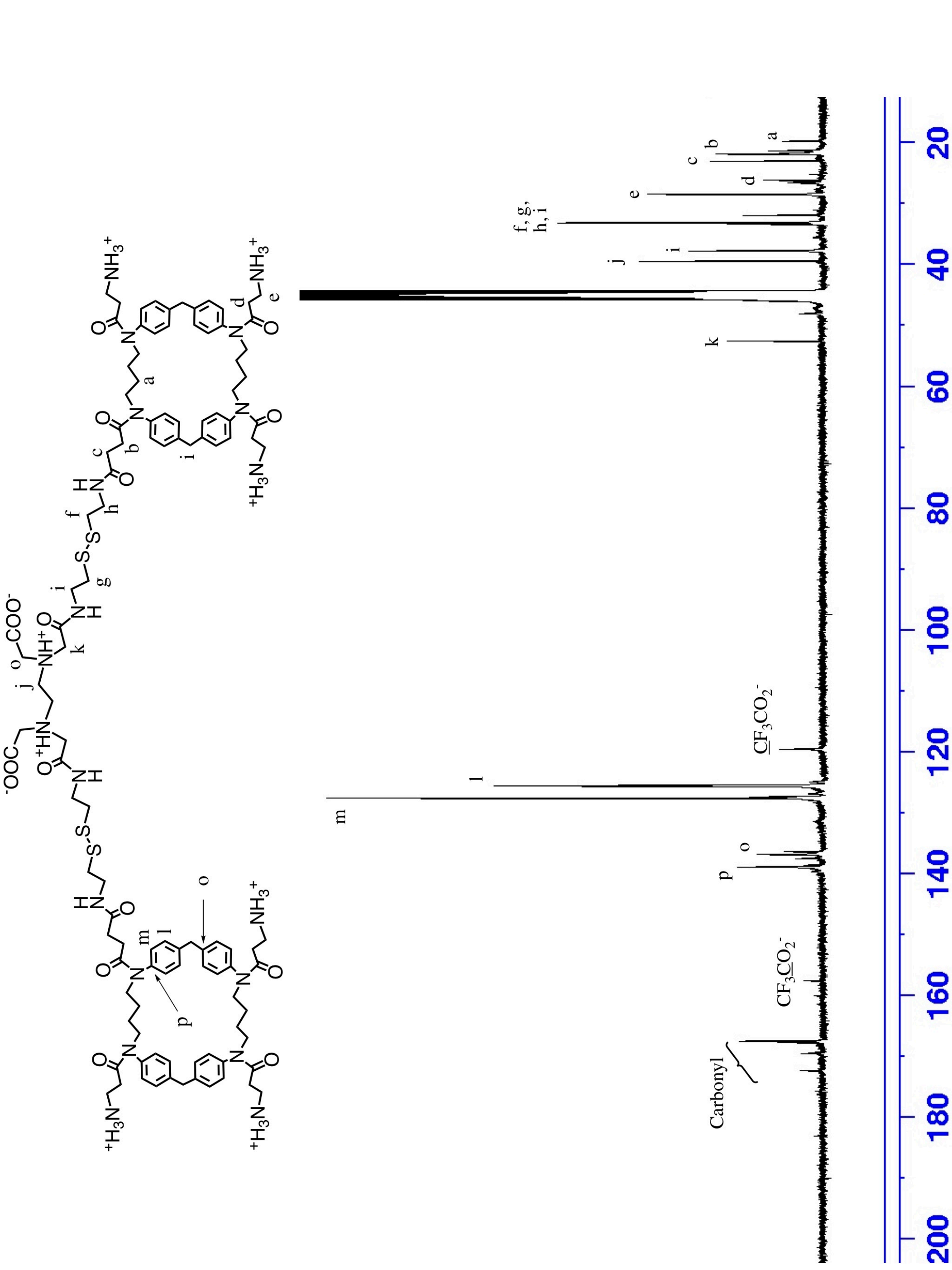

Figure S5. ^{13}C NMR spectrum of compound **1** (100 MHz, CD_3OD, 298K).

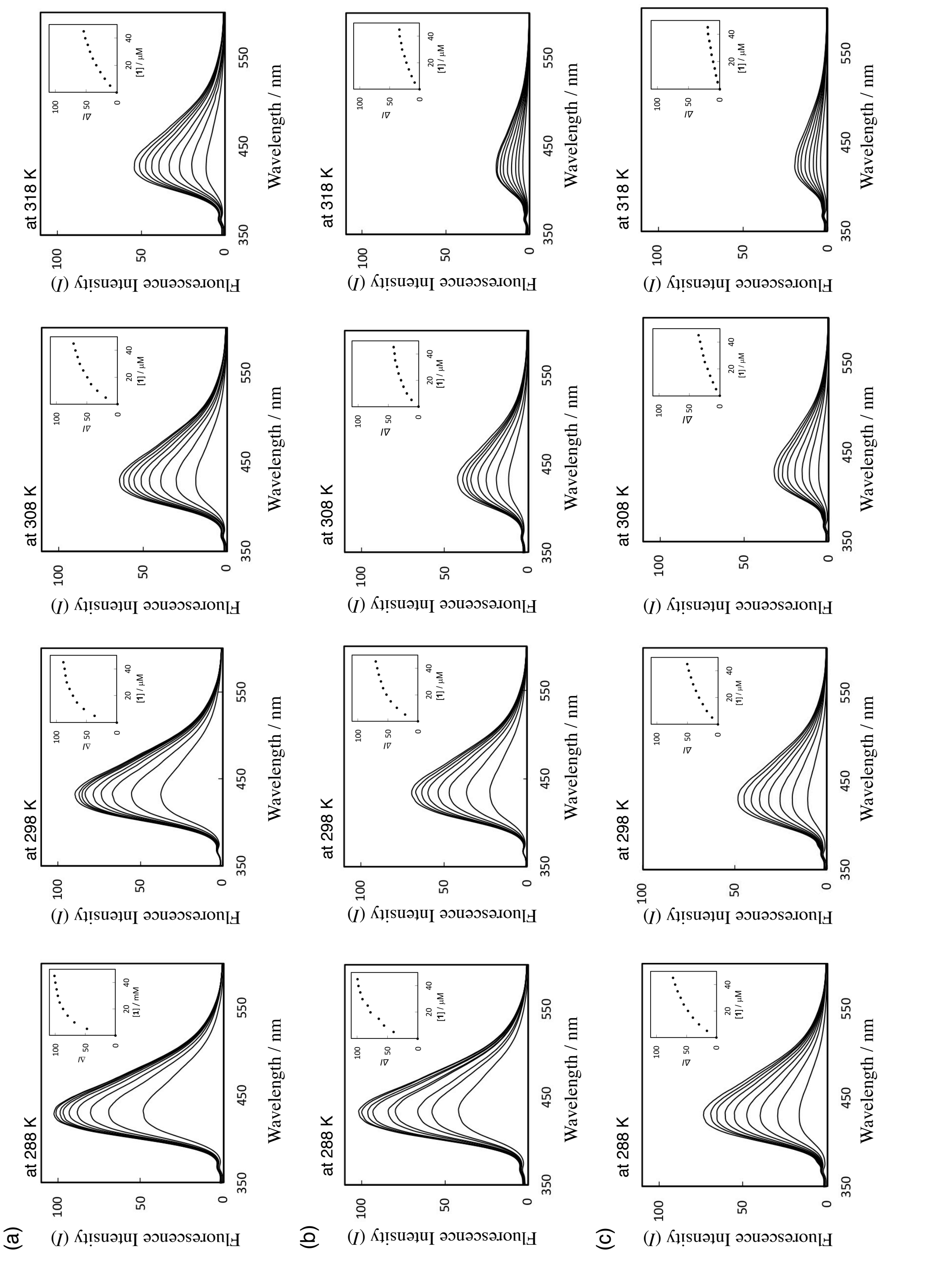

Figure S6. Fluorescence spectra for aqueous of TNS (1.0 μM) upon addition of **1** in aqueous buffer at 288, 298, 308, 318 K at pH 3.8 (a), 7.4 (b), and 10.7 (c). [**1**] = 0, 5, 10, 15, 20, 25, 30, 35, 40, and 45 μM. Ex. 326 nm.

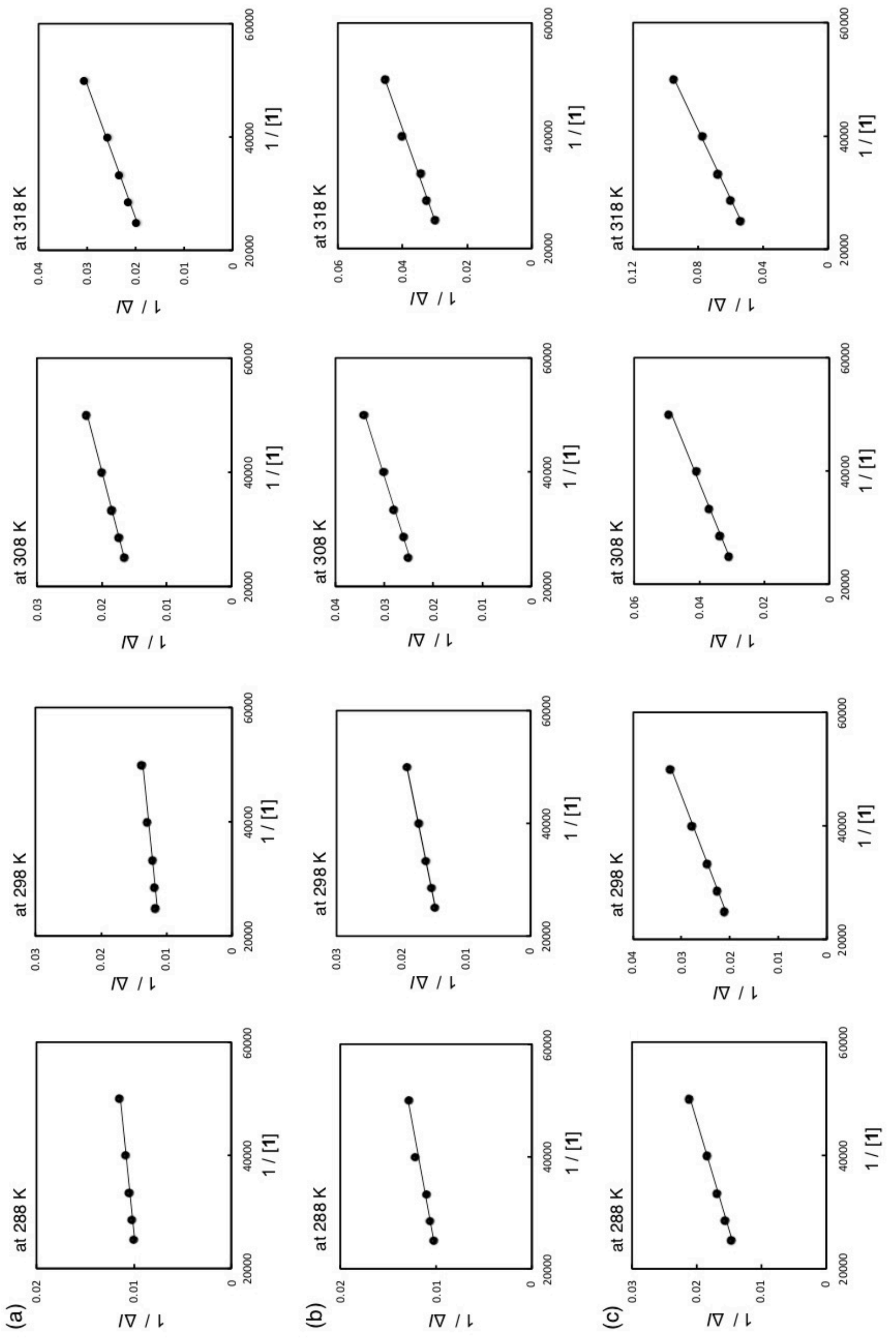

Figure S6 (continued). The corresponding double-reciprocal plots of the extent of change in fluorescence intensity against the total concentration of **1** at 288, 298, 308, 318 K at pH 3.8 (a), 7.4 (b), and 10.7 (c).

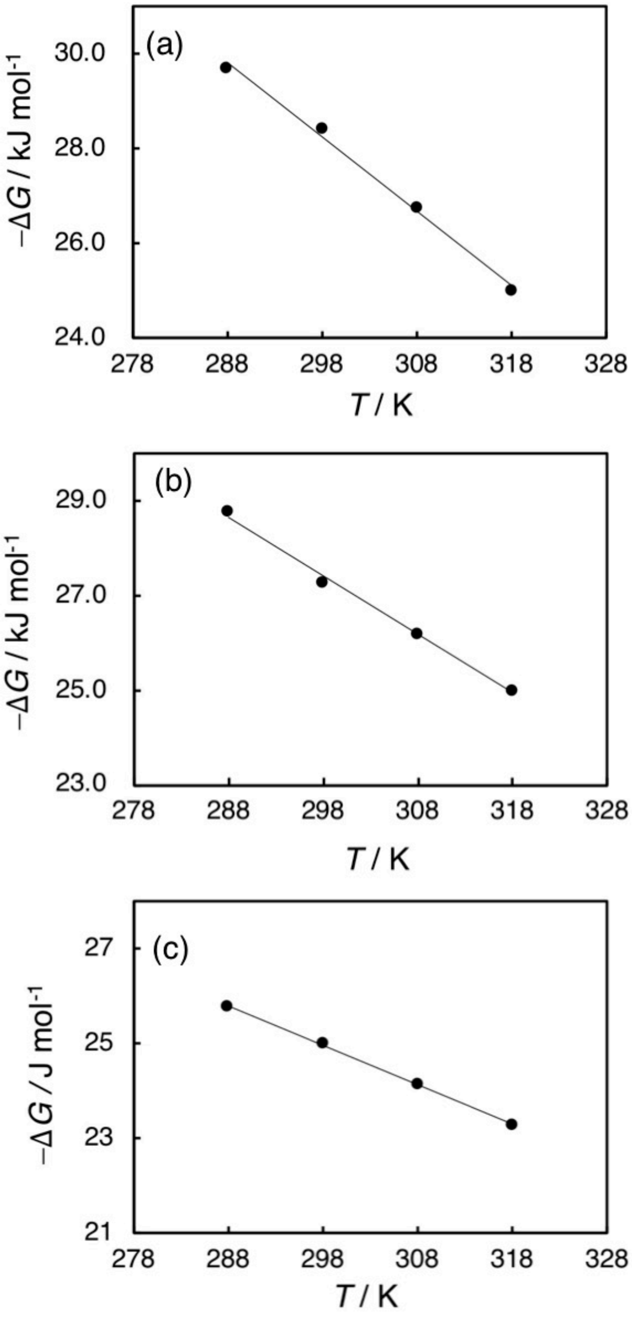

Figure S7. van't Hoff plots at pH 3.8 (a), 7.4 (b), and 10.7 (c)

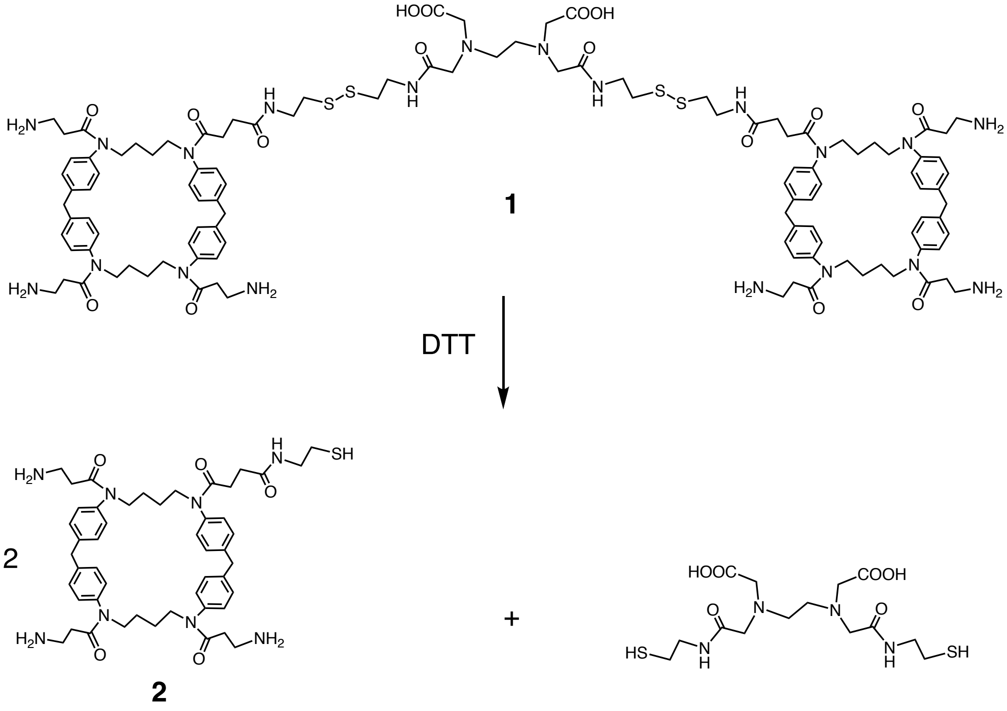

Thiol derivative of cyclophane
M denotes, $C_{49}H_{64}N_8O_5S$

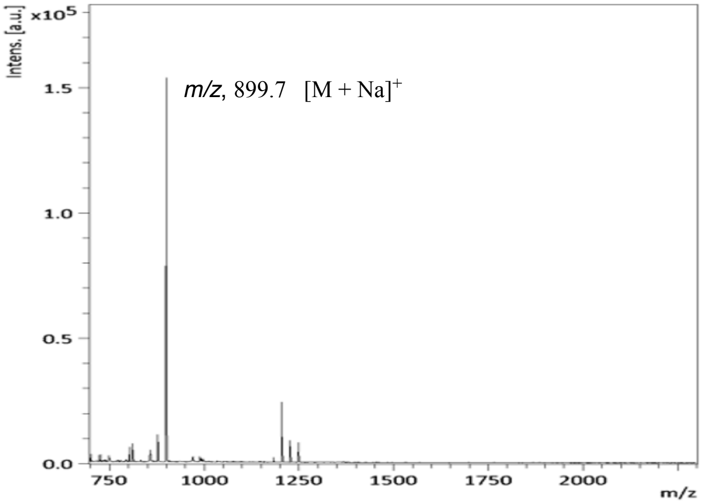

Fig. S8. MALDI-TOF MS spectra of **1** in the presence of DTT. Detection of the peaks originated from the thiol derivative of EDTA was difficult due to fragments of matrix with molecular weights less than 500 Da.

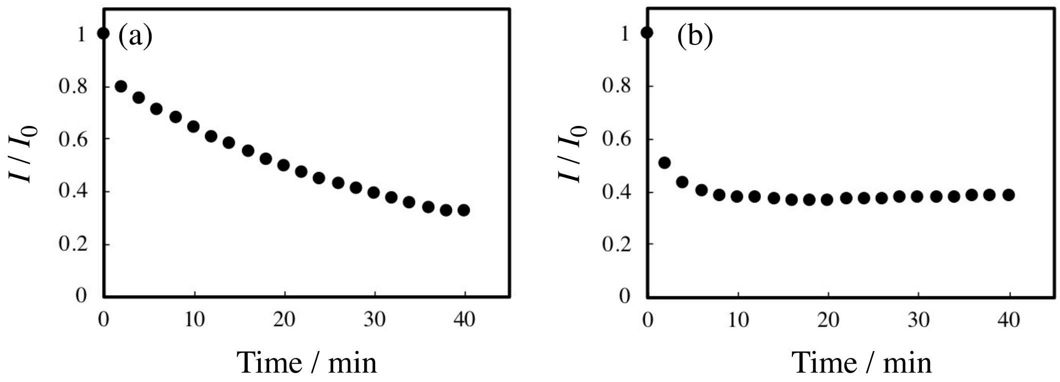

Figure 9*.* Time course for changes of fluorescence intensity originating TNS (1.0 μM) in the presence of **1** (25 μM) upon addition of GSH (50 μM) in aqueous buffer at pH 7.4 (a) and 10.7 (b)
